# French Toilet Preparations

**Published:** 1888-02

**Authors:** 


### FRENCH TOILET PREPARATIONS.

In a report submitted to the Hygienic Council of Paris, by Drs Dubrisay and Chafin, the authors state that the perfumery and toilet products now sold contain so many noxious substances that it is desirable the factories should be placed under special surveillance. They give a number of instances in support of their statement. The so-called "harmless and purely vegetable" hair dyes, they say, are all poisonous. "Progressive dyes" are ammoniacal solutions of nitrate of silver. The "instantaneous dyes" are a solution of litharge in lime water. "Eau des Fées" is a solution of sulphate of lead in hyposulphite of soda. "Eau Figaro" consists of three solutions: (1) of nitrate of silver and sulphate of copper; (2) sulphide of sodium; (3) cyanide of potassium (to remove the silver stains). "Eau des Fleurs" is composed of rose water 95.5; flowers of sulphur, 2.7; acetate of lead, 2.8. Passing to cosmetics, they say "Lait antiplelique" is composed of corrosive sublimate, 1.7; oxide of lead, 4.22; sulphuric acid and camphor. "Lait de Manille" is a mixture of borax, copper, tincture of benzoin, and essence of bitter almonds; "Lait de Ninon," of bismuth and zinc; "Eau Magique," oxide of lead and hyposulphite of zinc; "Eau de fleur de lys," protochloride of mercury; "Eau royal de Windsor," glycerine and oxide of lead; "Eau de Castille," hyposulphite of soda and acetate of lead. The "Poudre Pilivore de Laforet" contains mercury (?), 60 grs.; sulphide of arsenic, 30 grs.; litharge, 30 grains., and starch, 30 grs. "Epiteine" is simply sulphite of calcium, and "Antibolbos" hypophosphite of soda. Pomades against baldness all contain cantharides and croton oil.

